# A shared genetic basis of mimicry across swallowtail butterflies points to ancestral co-option of *doublesex*

**DOI:** 10.1038/s41467-019-13859-y

**Published:** 2020-01-03

**Authors:** Daniela H. Palmer, Marcus R. Kronforst

**Affiliations:** 1 Committee on Evolutionary Biology, University of Chicago, Chicago, IL 60637 USA; 2 Department of Ecology and Evolution, University of Chicago, Chicago, IL 60637 USA; 3 Present Address: Department of Animal and Plant Sciences, Alfred Denny Building, University of Sheffield, Western Bank, Sheffield, S10 2TN UK

**Keywords:** Evolutionary genetics, Mimicry

## Abstract

Uncovering whether convergent adaptations share a genetic basis is consequential for understanding the evolution of phenotypic diversity. This information can help us understand the extent to which shared ancestry or independent evolution shape adaptive phenotypes. In this study, we first ask whether the same genes underlie polymorphic mimicry in *Papilio* swallowtail butterflies. By comparing signatures of genetic variation between polymorphic and monomorphic species, we then investigate how ancestral variation, hybridization, and independent evolution contributed to wing pattern diversity in this group. We report that a single gene, *doublesex (dsx)*, controls mimicry across multiple taxa, but with species-specific patterns of genetic differentiation and linkage disequilibrium. In contrast to widespread examples of phenotypic evolution driven by introgression, our analyses reveal distinct mimicry alleles. We conclude that mimicry evolution in this group was likely facilitated by ancestral polymorphism resulting from early co-option of *dsx* as a mimicry locus, and that evolutionary turnover of *dsx* alleles may underlie the wing pattern diversity of extant polymorphic and monomorphic lineages.

## Abstract (web summary)

Polymorphic mimicry in *Papilio* swallowtail butterflies is thought to have had multiple independent origins. Here, the authors show that the gene *doublesex* controls mimicry across multiple species, but with distinct alleles that may have originated from an ancestral polymorphism.

## Introduction

An array of convergent adaptations are found across animal and plant taxa, arising from the interplay of natural selection, constraint, and historical contingency^1^. Comparing the genetic basis of convergent traits has been central to uncovering the genomic patterns and evolutionary processes that drive adaptation and phenotypic diversification^2^. By leveraging natural experiments of convergent adaptation, we are able to assess the extent to which evolution proceeds with shared genetic architecture, genes, and/or mutations. Furthermore, comparing how these evolutionary changes unfold in the genome among multiple taxa can uncover how ancestral variation, hybridization, or independent evolutionary trajectories contribute to adaptive phenotypes^3^.

Mimicry is a major convergent adaptation, and a significant driver of phenotypic evolution across animals and plants^4^. Much of our knowledge about the molecular foundations and evolution of mimicry has stemmed from research on wing pattern mimicry in butterflies^5–11^, a fundamental adaptation known to involve a diverse set of genes and genetic architectures. Perhaps the most extreme version of this adaptation is found in polymorphic mimicry, where discrete mimetic phenotypes co-occur within a species. Polymorphic mimicry has evolved in multiple butterfly lineages^9,12,13^, providing an opportunity to investigate the genetics and evolutionary origins of this complex adaptation. In *Heliconius numata*, polymorphic mimicry is controlled by a cluster of wing patterning genes contained within a chromosomal inversion^9,14^. This mimicry supergene along with other mimicry alleles are the product of frequent introgressive hybridization in the *Heliconius* clade^10,14^. In the genus *Papilio*, where polymorphic mimicry is typically limited to females, several independent molecular origins have been implicated in the evolution of wing pattern mimicry. Classic genetic crossing experiments on various *Papilio* species showed that either sex-linked or autosomal loci can function as ‘switches’ between female morphs^5,6,8^, and more recent molecular work has identified and characterized some of these mimicry loci^11,15–17^. In each of these studies, the mimicry loci of different *Papilio* species are located in distinct regions of the genome, with seemingly independent functional bases. These single-species analyses have illustrated the diverse molecular underpinnings generating polymorphic wing patterns. However, a comparative analysis of multiple polymorphic and monomorphic lineages is needed to elucidate the relative contributions of historical contingency and contemporary allele sharing to the evolution of mimicry and wing pattern diversity in this group.

Polymorphic mimicry is inferred to have evolved independently in several *Papilio* lineages^12,13^, but questions remain about the potential roles of ancestral variation and contemporary allele sharing in shaping the extant diversity of *Papilio* wing pattern phenotypes. Multiple *Papilio* species are known to share mimicry supergene architecture^5,7,8^, in which wing pattern variation is transmitted as a single Mendelian locus. More recently, studies in both *P. polytes* and *P. memnon* characterized their mimicry loci as having the same identity, the autosomal gene *doublesex* (*dsx*)^11,17–19^. The *dsx* mimicry alleles are distinct between *P. polytes* and *P. memnon*^18,19^, but without knowledge of *dsx* variation among other closely related species it is uncertain whether *dsx*-mediated mimicry arose de novo or as a product of shared ancestral variation. The extent to which *dsx* mediates polymorphic mimicry in other *Papilio* lineages, and how variation at *dsx* is related between polymorphic and monomorphic lineages are thus open questions that are pivotal to understanding the roles of genetic potentiation, constraint, and shared ancestry in mimicry evolution.

**Fig. 1:**
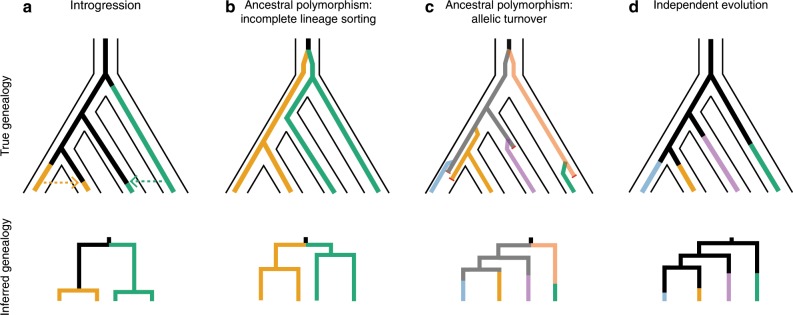
True (top row) and inferred (bottom row) genealogies under four mimicry evolution hypotheses. Colors represent distinct mimicry alleles/phenotypes. **a** Mimicry alleles are shared because of introgressive hybridization. **b** Mimicry evolves ancestrally, and descendant lineages inherit and maintain the ancestral alleles. **c** Mimicry evolves ancestrally, and alleles are gained and lost so that the extant alleles are distinct from the ancestral alleles. **d** Unique mimicry alleles arise by the independent evolution of mimicry. Under introgression and incomplete lineage sorting (ILS), the inferred genealogies will show alleles grouping by phenotype rather than species (**a**, **b**), but the branch lengths will be relatively shorter for introgression than for ILS. Under allelic turnover and independent evolution, the inferred genealogies will result in alleles grouping by species (**c**, **d**), but the branch lengths are expected to be longer for allelic turnover because of underlying balancing selection. A history of allelic turnover will also leave behind population genetic signatures of balancing selection, such as elevated Tajima’s D in the region of interest. Under allelic turnover, the amount of shared ancestral variation should track phylogenetic relatedness, because recombination and gene conversion will erase the evidence of ancestral variants as a lineage ages. Because shared ancestral variation is more likely to be retained at younger timescales, allelic genealogies may show alleles of one species nested within another species for younger lineages. In contrast, independent evolution need not involve balancing selection, and if variants are shared among lineages, these should not be correlated with the lineages’ phylogenetic proximity.

In this study, we test for convergence in the genetic basis of mimicry among four closely related female polymorphic lineages, *P. polytes*, *P. memnon*, *P. rumanzovia*, and *P. aegeus*. We test whether the evolution of polymorphic mimicry involves changes at the same genomic location, and we find that the same gene, *dsx*, controls female polymorphic mimicry in all four species. We then analyze closely related monomorphic lineages to infer the historical processes driving mimicry evolution and wing pattern diversity across this group. Specifically, we explore four hypotheses for the evolution of mimicry in this clade: (1) introgression, (2) ancestral polymorphism with incomplete lineage sorting, (3) ancestral polymorphism with allelic turnover, and (4) independent evolution (Fig. 1). In (1), mimicry alleles are transmitted between hybridizing lineages by introgression (Fig. 1a). In (2), polymorphic mimicry evolves ancestrally and the ancestral mimicry alleles are inherited and maintained by multiple lineages through balancing selection (Fig. 1b). In (3), polymorphic mimicry evolves ancestrally, but mimicry alleles are subsequently replaced by their own allelic descendants, or lost by drift, a process known as allelic turnover^20–22^ (Fig. 1c). In (4), mimicry alleles arise independently in each lineage by repeated co-option of *dsx* (Fig. 1d). Our results indicate the presence of distinct *dsx* mimicry alleles among species, ruling out both introgression and incomplete lineage sorting. While we cannot rule out the possibility of independent *dsx* co-option, we do find evidence consistent with allelic turnover. Independent evolution and allelic turnover are expected to result in distinct alleles among species, but allelic turnover will produce allelic genealogies with elongated branches due to underlying multiallelic balancing selection^22,23^. Similarly, population genetic signatures associated with balancing selection can be used to differentiate independent evolution from allelic turnover. Together with previous work, our findings suggest that ongoing turnover of *dsx* alleles from a polymorphic ancestor underlies much of the wing pattern diversity in this group. These findings imply a dynamic trajectory for mimicry evolution, potentially involving many gains and losses of mimicry alleles.

## Results

### Mimicry loci in *P. rumanzovia*, *P. memnon*, *P. aegeus*

We first identified the mimicry loci of *P. rumanzovia*, *P. memnon*, and *P. aegeus* using genome-wide association studies (GWAS) and principal component analyses (PCA) of single nucleotide polymorphism (SNP) data. We analyzed female specimens representing three *P. rumanzovia* morphs (simple, blended, white), two *P. memnon* morphs (band, patch), and two *P. aegeus* morphs (light, dark).

**Fig. 2:**
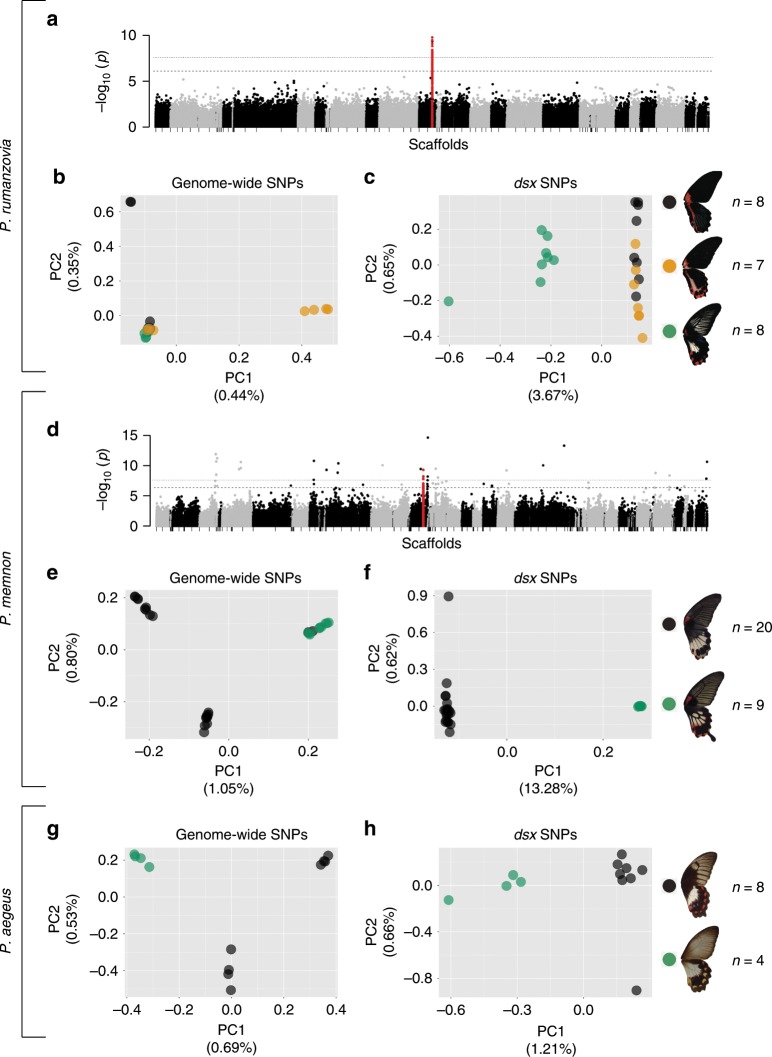
Identifying mimicry loci in *P. rumanzovia*, *P. memnon*, and *P. aegeus*. Genome-wide association study (GWAS) for wing patterning in *P. rumanzovia* (**a**) and *P. memnon* (**d**). Principal component analysis (PCA) based on genome-wide variants for *P. rumanzovia* (**b**), *P. memnon* (**e**), and *P. aegeus* (**g**). PCA based on *dsx* variants for *P. rumanzovia* (**c**), *P. memnon* (**f**), and *P. aegeus* (**h**). Red points in **a** and **d** indicate SNPs in the *dsx* region. Dashed and dotted lines in **a** and **d** show the false discovery rate (*q*-value) cutoffs of 0.01 and 0.001, respectively.

In *P. rumanzovia*, a GWAS with the three morphs and 869,047 variants revealed a single peak of association positioned within the *dsx* gene (Fig. 2a). In additional GWAS comparing pairs of morphs we observed the same peak of variants in the *dsx* region for the simple/white and blended/white comparisons, but not for the simple/blended (Supplementary Fig. 1). For the simple/blended GWAS, only one highly associated variant remained within the *dsx* region, and several emerged in the vicinity of *dsx* (Supplementary Fig. 1C). We next sought to characterize the population substructure associated with *dsx* mimicry alleles using methods originally developed to identify chromosomal inversions from SNP data^24^. We compared population structure to local *dsx* genotypes using principal component analysis (PCA) based on genome-wide SNPs and PCA based on *dsx* SNPs. The genome-wide PCA based on ~2.5 million SNPs placed most individuals into a single cluster (Fig. 2b). In contrast, the *dsx* PCA for the same individuals based on 779 *dsx* -specific SNPs resulted in the white individuals separating from the simple and blended individuals along PC1 (Fig. 2c). Most of the white individuals were found to be heterozygous at *dsx*, resulting in their intermediate placement along PC1 (Fig. 2c). These results suggest that variation at the *dsx* locus differentiates the *P. rumanzovia* white patch morph from the simple and blended morphs, and that the simple and blended morphs may be determined by variation in and around *dsx*.

The GWAS for *P. memnon* using approximately 1.8 million variants showed noise in genome-wide association values, but the densest cluster of highly associated variants fell within the *dsx* region (Fig. 2d). The genome-wide PCA (~2.5 million SNPs) revealed that white band morphs differ from white patch morphs across the genome, possibly due to different geographic origins of our band and patch samples. This causes variants across the genome to appear highly associated with wing pattern phenotype (Fig. 2d), when in reality these variants are unrelated to wing patterning. The PCA for the same set of individuals based on 644 *dsx*-specific SNPs segregated individuals by wing pattern along PC1 (Fig. 2f). These results point to *dsx* as the mimicry locus in *P. memnon*, consistent with other recent works on *P. memnon*^18,19^.

We were not able to generate GWAS results for *P. aegeus* due to low sample size. The PCA for approximately 1.5 million genome-wide SNPs revealed differentiation between light and dark morphs across the genome, as in *P. memnon* (Fig. 2g), but the PCA with only *dsx* SNPs (500 SNPs) showed individuals segregating by phenotype along PC1 (Fig. 2h). Like in *P. rumanzovia*, the light morph individuals were nearly all *dsx* heterozygotes and fell in the center of PC1 (Fig. 2h). From these results, we concluded that *dsx* is also associated with polymorphic wing patterning in *P. aegeus*.

### Genetic differentiation and linkage disequilibrium at *dsx*

**Fig. 3:**
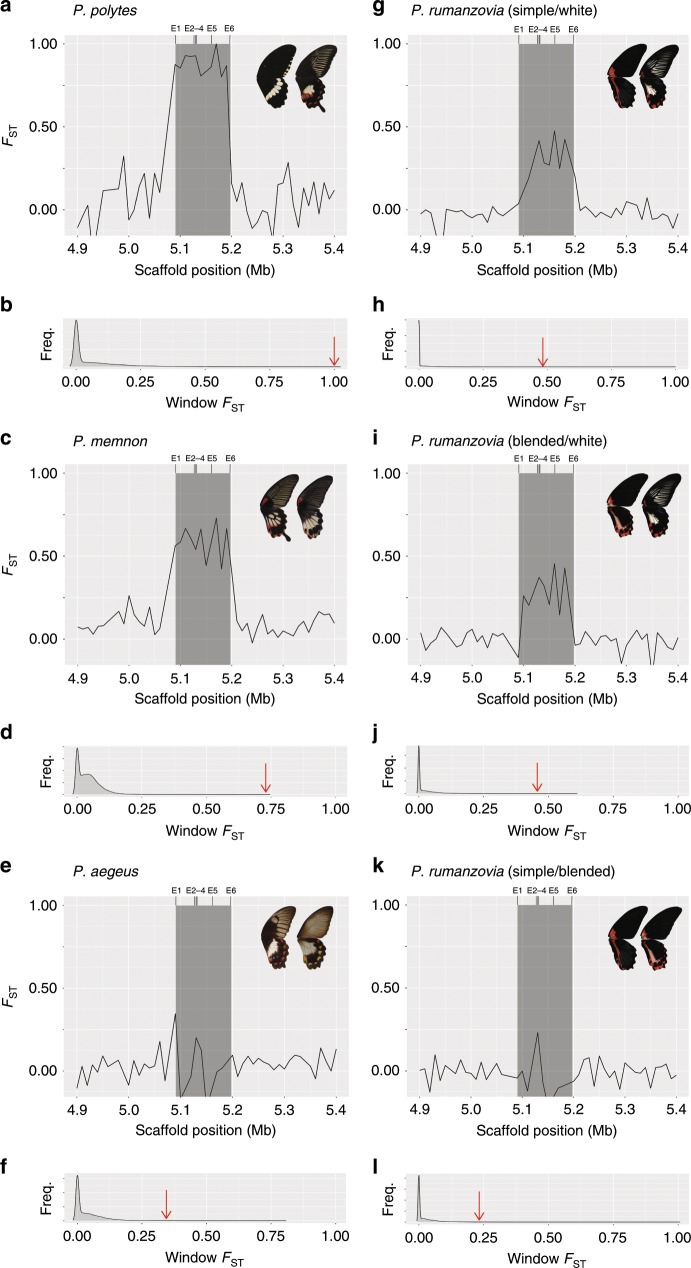
Genetic differentiation between morphs. *F*_*ST*_ across a 500 kb interval containing *dsx* (gray box) in *P. polytes* (**a**), *P. memnon* (**c**), *P. aegeus* (**e**), and *P. rumanzovia* (**g**, **i**, **k**). E1–6 indicate the position of *dsx* exons. Genome-wide *F*_*ST*_ distribution in *P. polytes* (**b**), *P. memnon* (**d**), *P. aegeus* (**f**), and *P. rumanzovia* (**h**, **j**, **l**). Red arrows indicate the highest *F*_*ST*_ value for *dsx* windows, all of which are in the 99th percentile of their respective distributions.

We calculated *F*_*ST*_ between wing pattern morphs within each polymorphic species to analyze patterns of genetic differentiation across the mimicry locus *dsx*. For each comparison, we applied a 10 kb window size across the genome and removed windows with a low number of variants (bottom 10% by species). We visualized the genetic differentiation between morphs across the *dsx* region and compared these values to the genome-wide *F*_*ST*_ distribution. First, we calculated *F*_*ST*_ between the mimetic and non-mimetic *P. polytes* female morphs as a positive control for highly divergent, inverted *dsx* haplotypes^11,17^. We observed a plateau of elevated *F*_*ST*_ across the entire *dsx* region, reflecting the inverted chromosomal structure of *dsx* in *P. polytes* (Fig. 3a). Furthermore, the highest *F*_*ST*_ value for *dsx* windows was in the 99th percentile of the genome-wide distribution of *F*_*ST*_ values (Fig. 3b). In subsequent comparisons for *P. rumanzovia*, *P. memnon*, and *P. aegeus*, each species showed elevated *F*_*ST*_ at *dsx* relative to the rest of the genome, but we observed species-specific patterns of differentiation within the *dsx* region. For *P. rumanzovia*, we calculated *F*_*ST*_ between the three pairs of morphs (simple/white, blended/white, simple/blended) using the 23 individuals from the PCAs. The results showed high *F*_*ST*_ for *dsx* windows in comparisons between simple/white and blended/white, consistent with the GWAS and PCA results, but this region of elevated *F*_*ST*_ did not include exon one (Fig. 3g, i). The highest *F*_*ST*_ value for *dsx* windows in each of the simple/white and blended/white comparisons was also in the 99th percentile of their respective genome-wide *F*_*ST*_ distributions (Fig. 3h, j). Between the simple and blended morphs, only one *dsx* window showed elevated *F*_*ST*_, driven by the single highly associated SNP we observed in the GWAS (Fig. 3k, Supplementary Fig. 1C). In *P. memnon*, the entire length of *dsx* showed elevated *F*_*ST*_ between the 20 band individuals and 9 patch individuals, resembling the plateau observed across *dsx* in *P. polytes* (Fig. 3c). For *P. aegeus*, the elevated *dsx F*_*ST*_ was restricted to the window containing exon one and the window containing exon four (Fig. 3e). The highest *F*_*ST*_ value for *dsx* in both *P. memnon* and *P. aegeus* was in the 99^th^ percentile of their respective genome-wide distributions, consistent with earlier GWAS and PCA (Fig. 3d, f).

**Fig. 4:**
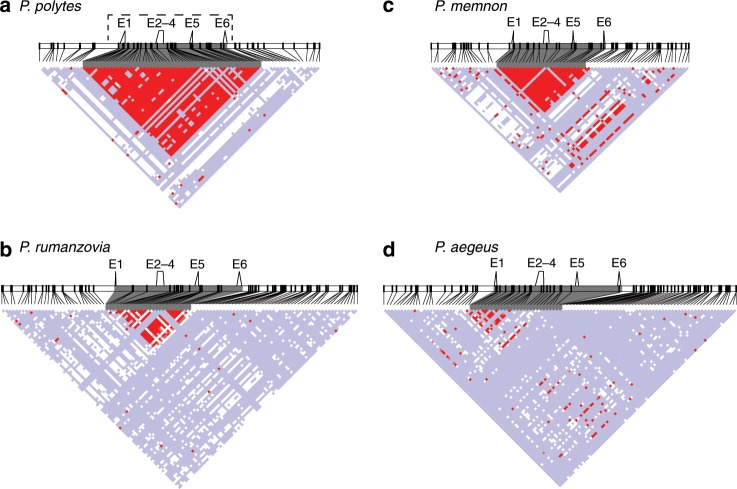
Linkage disequilibrium (LD) heat maps. LD across *dsx* (gray shading) and flanking 100 kb for *P. polytes* (**a**), *P. rumanzovia* (**b**), *P. memnon* (**c**), and *P. aegeus* (**d**). Standard color scheme: D’ < 1, LOD < 2 (white); D’ = 1, LOD < 2 (blue); D’ ≤ 1, LOD ≥ 2 (pink and red). Dashed line in **a** shows the inverted region. E1–6 indicate the *dsx* exons.

We next analyzed patterns of linkage disequilibrium (LD) within *dsx* and the surrounding region for each species, again using *P. polytes* as a positive control for highly differentiated mimicry haplotypes. *P. polytes* showed high LD across the entire length of *dsx*, consistent with prior analyses and the known inversion spanning the *dsx* region^17^ (Fig. 4a). In *P. rumanzovia*, a subset of the *dsx* region spanning exons two through six showed elevated LD, reflecting the region of elevated *F*_*ST*_ described above (Figs. 4b, 3g, i). For *P. memnon* we observed a region of elevated LD across the length of *dsx* like in *P. polytes*, corroborating the high *F*_*ST*_ plateau across this region (Figs. 4c, 3c). The elevated LD signature for *dsx* in *P. aegeus* was localized to a region containing exon one, which was also consistent with the *F*_*ST*_ results (Figs. 4d,  3e).

### De novo genome assembly in *P. rumanzovia*

We generated de novo genome assemblies for *P. rumanzovia* to validate our methods and explore structural variation at *dsx*. We used combined mate-pair and paired-end sequencing datasets to assemble the genomes of one female that we inferred to be homozygous for the simple morph-associated haplotype and one female inferred to be homozygous for the white morph-associated haplotype. The assembled genome sizes for the simple and white morph were 218 and 215 Mb, respectively, which are similar to the 227 Mb *P. polytes* genome^11,17^. The scaffold N50 values for the simple and white assemblies were 53 kb and 197 kb, respectively. We identified four scaffolds in the simple morph assembly and three scaffolds in the white morph assembly that contained *dsx* exons (Supplementary Fig. 2). In the simple morph assembly, two scaffolds provided structural information. First, exons two through four were assembled on a single scaffold, at approximately the same distance and orientation to one another as in *P. polytes*. Second, exon six and the neighboring *ubiquitously expressed transcript* (UXT) were assembled together, revealing the collinearity of the simple morph with the non-mimetic *P. polytes* morph and with the outgroup taxon *P. xuthus*. With the white morph assembly, we could gather structural information from one scaffold which contained exons one through four arranged at approximately the same distance and orientation as in *P. polytes*. Our comparisons of the simple and white morph showed a sharp increase in *F*_*ST*_ and LD between exons one and two, and a drop in these signatures between exons five and six (Figs. 3g, 4b), which could be indicative of a chromosomal inversion in this region. This inversion would have to be in the simple morph, given that exons one through four coassembled onto a single  white morph scaffold. Together, these data show that if there exists a *dsx* inversion in *P. rumanzovia*, its breakpoints are quite different from those of *P. polytes*.

We aligned the scaffolds containing *dsx* exons to further explore sequence differentiation between the *P. rumanzovia* simple and white morphs (Supplementary Fig. 2). Consistent with the *F*_*ST*_ analysis (Fig. 3g), we generally observed higher sequence identity surrounding exon one and much lower identity for regions surrounding exons two through six. In contrast to the numerous substitutions between the *P. polytes* mimetic and non-mimetic across exons one through six, we found that the simple and white *P. rumanzovia* sequences differed only at exons five and six, and had identical sequences for exons one through four. Exon five, however, is only spliced into the male *dsx* isoform in *P. polytes*, and exon six is a non-coding exon^11^. While we cannot conclude what the functional impacts of these differences are, these results appear to indicate that the switching between female mimicry phenotypes in *P. rumanzovia* is not a result of *dsx* protein coding differences, but likely a regulatory phenomenon.

### Evolutionary relationships between *dsx* haplotypes

**Fig. 5:**
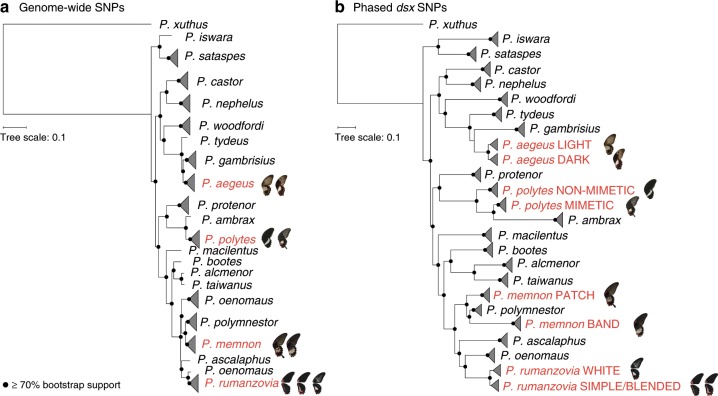
Phylogenetic relationships and evolution of mimicry in *Papilio*. Maximum-likelihood phylogenies for polymorphic (red) and monomorphic (black) *Papilio* butterflies based on genome-wide SNPs (**a**) and phased *dsx* SNPs (**b**).

In order to trace the evolutionary history of *dsx*-mediated wing pattern polymorphism we characterized the phylogenetic relationships of polymorphic and monomorphic *Papilio* species using both genome-wide SNPs and phased *dsx* SNPs. We generated a maximum-likelihood species tree based on ~3.4 million SNPs from the coding sequence of 51 individuals representing 16 monomorphic and 4 polymorphic species (Fig. 5a, Supplementary Data 1). Our topology was largely consistent with published phylogenies^13,25,26^, but included taxa that had not been sampled in previous phylogenies. We then phased approximately 7000 SNPs from the *dsx* region and built a maximum-likelihood gene tree for 116 individuals (Supplementary Data 1). The *dsx* gene tree topology mirrored the species tree topology, with the polymorphic haplotypes clustering by species (Fig. 5b). We observed no clustering of haplotypes between species indicative of *dsx* allele sharing.

We were surprised to find all mimicry haplotypes clustering by species in the *dsx* gene tree, with no apparent allele clustering between species. However, ongoing recombination or gene conversion between *dsx* haplotypes within species could erode signatures of allele sharing due to ancestry or hybridization. Gene conversion occurs when one allelic sequence is copied onto its homolog during DNA repair, resulting in homogenization of alleles over lengths of 100–2000 bp^27^. We tested for gene conversion between *dsx* haplotypes within each polymorphic species using GENECONV^28^ and found two significant tracts of identical sequence (Supplementary Table 1). The first was in *P. polytes*, and coincided with a region of decreased *F*_*ST*_ (Supplementary Table 1, Fig. 3a). The second putative gene conversion tract was between the *P. rumanzovia* simple and white morphs, and also coincided with the low *F*_*ST*_ and LD signatures observed for that part of the *dsx* region (Supplementary Table 1, Fig. 3g, Fig. 4b). However, given the extended lengths of these putative tracts compared to typical gene conversion events, these regions appear to reflect areas of recombination between *dsx* alleles.

**Fig. 6:**
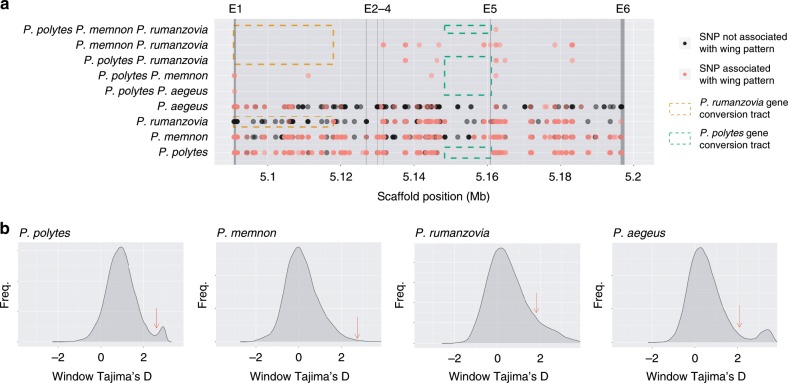
Trans-species polymorphisms and evidence of balancing selection at *dsx*. **a** Unique and shared SNPs across the *dsx* (gray box) region. Vertical boxes E1–6 indicate the *dsx* exons. Color variation in dots is due to overlapping points. Species combinations not shown had no shared SNPs. **b** Genome-wide Tajima’s D distributions. Red arrows indicate the highest Tajima’s D value for *dsx* windows.

While our phylogenetic analysis showed that *dsx* haplotypes were not shared among species, we could expect to find some trans-species polymorphisms associated with wing patterning across species if *dsx* haplotypes were inherited from a polymorphic ancestor. We identified SNPs associated with wing patterning in each polymorphic species, and then compared these across all species to assess if any SNPs were shared among taxa. There were some shared SNPs among species, but the number was small (1–19 SNPs), and we observed that only some combinations of species shared associated polymorphisms (5/11 comparisons; Fig. 6a). The number of shared SNPs was higher in more phylogenetic proximate taxa, with *P. rumanzovia* and *P. memnon* sharing the most variants (Fig. 6a). As expected, there were virtually no SNPs associated with wing pattern in the respective gene conversion/recombination tracts of *P. polytes* and *P. rumanzovia*, and therefore no shared SNPs within these regions. This suggests that any ancestrally shared variants in these regions would have been eroded away by gene conversion and recombination, thus erasing the expected phylogenetic signature of ancestral polymorphism.

To further explore the scenario of ancestral polymorphism, we calculated Tajima’s D for *dsx*-specific variants and compared these to the genome-wide distribution of Tajima’s D values for each polymorphic lineage. In each species, Tajima’s D was elevated for *dsx* variants (Fig. 6b). Peak Tajima’s D values for the *dsx* region were in the 96^th^ percentile for *P. polytes*, the 87^th^ for *P. rumanzovia*, the 99^th^ for *P. memnon*, and the 91^st^ for *P. aegeus*.

## Discussion

Our study reveals a dynamic history of mimicry and wing pattern evolution within *Papilio* that involves changes at a single autosomal gene, *dsx*. Although we find the same gene underlying polymorphic mimicry in multiple taxa, our analyses reveal species-specific patterns of genetic variation and linkage disequilibrium between *dsx* mimicry alleles. In *P. polytes* the mimetic and non-mimetic *dsx* haplotypes differ by thousands of substitutions in their coding and non-coding sequence, and an inversion spans the entire *dsx* gene, possibly affecting neighboring gene function^11,17,26^. In contrast, *P. rumanzovia* showed an extended region of genetic differentiation and LD across *dsx* exons two through six, but only distinguishing the white morph from the simple and blended morphs. Exon one, which codes for most of the Dsx protein and an essential DNA binding domain, is not differentiated between morphs. Furthermore, we found that the differences in *dsx* sequence were almost exclusively in the non-coding regions of the gene. Together these patterns suggest that variation in *dsx* regulation controls mimicry in *P. rumanzovia*, echoing previous work suggesting that *dsx* coding differences do not contribute to mimicry in *P. polytes*^26^. In *P. memnon* we observed signatures more similar to *P. polytes*, including sequence differences and high LD between morphs across the entirety of *dsx* and substitutions in both coding and non-coding sequence. Finally, in *P. aegeus* we observed differentiation between morphs in coding and non-coding sequence and high LD concentrated around exon one. While we cannot pinpoint the mutations that drive female development into alternative morphs, these results suggest that there is not a shared functional mechanism underlying *dsx* -mediated mimicry in different *Papilio* species.

Recombination between *dsx* alleles is reduced to some degree in each polymorphic species, but reduced recombination appears to be caused by different mechanisms. In contrast to the chromosomal inversion spanning *dsx* in *P. polytes*, Iijima et al.^19^ found no rearrangement between the *P. memnon dsx* alleles. Instead, they suggest that large indels and repetitive sequences are responsible for suppressing recombination between *dsx* alleles in *P. memnon*. Our de novo assembly similarly indicated that the *P. polytes* inversion breakpoints were not present in *P. rumanzovia*, although there still exists the possibility that a smaller inversion could be contained within the *dsx* region. The *dsx* inversion may thus be a feature specific to the *P. polytes* lineage.

Convergent *dsx*-mediated mimicry could arise by different evolutionary processes including introgression, incomplete lineage sorting, allelic turnover, and/or independent evolution (Fig. 1). We found that *dsx* mimicry haplotypes are unique among taxa, inconsistent with the expected signatures of introgression or incomplete lineage sorting. While the expected signatures of introgression and incomplete lineage sorting involve clear discrepancies between gene and species trees, the scenarios of allelic turnover and independent evolution are more difficult to distinguish from one another. Allelic turnover is another outcome of ancestral polymorphism, but involves a variety of processes that result in distinct ancestral and extant alleles. Takahata^20^ described this scenario in the context of the major histocompatibility complex, in which polymorphic alleles of different functional classes (analogous to wing pattern haplotypes in our system) are shared among species and maintained by balancing selection, or lost by genetic drift. In an allelic turnover event, an existing allele mutates to a new descendant allele, which then randomly replaces an existing allele. Thus, new alleles are continuously produced, and eventually replace the ancestral alleles. Adding recombination to this model effectively amplifies the effects of allelic turnover, as nucleotides are shuffled among existing alleles^29^. Allelic turnover and recombination/gene conversion will thus progressively erode trans-species polymorphisms that may have been present in the past.

While aspects of the allelic turnover model are similar to normal molecular divergence, there are some clear distinctions that suggest *dsx* is likely evolving according to the allelic turnover model in *Papilio*. For instance, both allelic turnover and normal molecular divergence are expected to result in an allelic genealogy structure that matches the basic coalescent^20,22^. However, because allelic turnover is the product of multiallelic balancing selection, the timescale of the coalescent is expected to be different, leading to an elongation of the allelic genealogy and long terminal branches in particular^22,23^. Consistent with these predictions, we found that the structure of the *dsx* tree largely matched the genome-wide tree but the branch lengths appeared to be longer (Fig. 5). There were also two instances in the *dsx* tree in which haplotypes from one species were nested within another species (*P. ambrax* within *P. polytes* and *P. polymnestor* within *P. memnon*, Fig. 5b), suggesting some retention of ancestral variation at younger time scales, as expected under the allelic turnover model. Furthermore, the elevated Tajima’s D statistic in the *dsx* region of each polymorphic lineage is also consistent with balancing selection. Previous research has also shown this turnover process play out on relatively short timescales. Zhang et al.^26^ showed that new mimicry alleles arose independently in each of two *P. polytes* subspecies in the course of approximately 1.7 MY, illustrating the rapid timescale of *dsx* turnover. They also revealed the loss of *dsx* polymorphism in *P. ambrax* and *P. phestus*, close relatives of *P. polytes* that fixed the mimetic allele and lost the non-mimetic allele due to drift and selection. These examples illustrate the gain and loss of *dsx* alleles in the *P. polytes* species group.

Although we observed phylogenetically distinct *dsx* haplotypes among species which could have arisen by independent evolution in each lineage, we also identified several trans-species *dsx* polymorphisms associated with wing patterning which may further indicate that this variation predates the origin of our focal taxa. If these shared polymorphisms are indeed the remnants of an ancestral *dsx* wing pattern polymorphism, the evidence of ongoing gene conversion and/or recombination between *dsx* haplotypes helps explain why there remains little shared variation and thus the haplotypes appear to be distinct between species. Among our comparisons, *P. memnon* and *P. rumanzovia* shared the most SNPs associated with wing pattern, and are also the two most closely related polymorphic species of the four. Comparisons involving *P. memnon*/*P. rumanzovia*, and *P. polytes* had the second highest number of shared polymorphisms. *P. aegeus*, the most distant relative of the other three, only shared one associated SNP with *P. polytes* (and zero with *P. memnon* or *P. rumanzovia*). This phylogenetic signal among the trans-specific polymorphism data, in which more closely related species share more wing pattern-associated variants, is consistent with the idea that these SNPs represent ancestral polymorphisms rather than being the product of recurrent mutation. Overall, our results are consistent with the predicted effects of allelic turnover and recombination/gene conversion on ancestral polymorphisms. Our findings thus indicate that the gain and loss of mimicry alleles may be widespread in shaping *Papilio* wing pattern diversity. Although previously regarded as an evolutionary dead-end^12,13,30^, mimicry in *Papilio* butterflies appears to be an ancient adaptation that has fueled the dynamic evolutionary turnover of wing patterning alleles.

## Methods

### Sample preparation and sequencing

130 adult butterflies were collected from the wild and from butterfly farms (Supplementary Data 1). Approximately 10 mg of thoracic tissue was removed from each individual and genomic DNA was extracted using a chloroform-based protocol. 100 bp paired-end libraries were prepared using the KAPA Hyper Prep Kit. *P. rumanzovia* libraries were sequenced on an Illumina HiSeq2500 and all others were sequenced on an Illumina HiSeq4000. Raw reads were demultiplexed based on their barcodes (Supplementary Data 1).

### Data collection and genotype calling

We chose to use the monomorphic outgroup species *P. xuthus* as the reference genome for all read-mapping instead of the more closely related *P. polytes* to avoid biasing our results with a polymorphic reference. We downloaded the *P. xuthus* v1.0 genome from PapilioBase^17^ and *P. polytes* resequencing data from three homozygous mimetic and three homozygous non-mimetic females from NCBI (SRR1118152, SRR1118150, SRR1118145, SRR1112619, SRR1112070, SRR1111718)^11^. Reads from the 136 total genome resequencing datasets were quality trimmed using SLIDINGWINDOW:4:15 in Trimmomatic^31^ and remaining reads were mapped to the *P. xuthus* v1.0 genome using the --very-sensitive-local option in Bowtie2^32^. Mapped reads were then re-ordered, sorted, and deduplicated with Picard (http://picard.sourceforge.net). We called variants using GATK’s^33^ HaplotypeCaller with options --emitRefConfidence GVCF --heterozygosity 0.01 -stand_call_conf 50.0 and performed joint genotyping using GenotypeGVCFs. For SNPs we filtered out the bottom 10% by quality and with FS > 60.0 and ReadPosRankSum λ−8.0, and for indels we filtered out the bottom 10% by quality and with FS > 200.0 and ReadPosRankSum <−20.0 using GATK’s VariantFiltration.

We became aware of the presence of microsporidian sequences (specifically of *Nosema bombycis*) in the *P. xuthus* genome assembly. We analyzed four representative datasets to verify that our results were not impacted by the presence of these sequences (see Supplementary Note 1).

### Genome-wide association and principal component analyses

VCF files containing SNP and indel calls were converted to PLINK format using VCFTools^34^. Phenotypes were assigned using a custom script and files were converted to GEMMA input using PLINK^35^. We used GEMMA^36^ to perform association tests between genotypes and wing pattern phenotype using option -miss 0.20. Benjamini-Hochberg false discovery rate (*q*-value)^37^ cutoffs of 0.01 and 0.001 were calculated for each GWAS in R^38^ and manhattan plots were generated using the qqman R package^38,39^. We used PLINK^35^ to perform PCA using only SNP calls and option --geno 0.1. PCA plots were generated using the ggplot2 package in R^38,40^.

### *F*_*ST*_ analysis

We calculated Weir and Cockerham’s *F*_*ST*_^41^ in 10 kb windows across the genome based on SNP calls using VCFTools^34^ with options --weir-fst-pop and --fst-window-size 10000. We calculated the distribution of the number of variants per window for each pairwise comparison and removed windows in the bottom 10% because these windows are more likely to show artificially extreme *F*_*ST*_ values. *F*_*ST*_ line graphs and distributions were plotted with the ggplot2 R package^38,40^.

### Linkage disequilibrium (LD) analysis

VCF files containing SNP calls across *dsx* and the flanking 100 kb were converted to Haploview format using PLINK^35^. A random subset of biallelic variants was selected using PLINK’s --thin option to yield ~1000 representative variants per species. We used Haploview^42^ to calculate pairwise LD between variants with a minimum genotyping rate of 75% and minimum minor allele frequency of 0.001. LD heat maps were exported from Haploview.

### De novo genome assembly

We used genomic DNA extracted for paired-end sequencing to generate mate-pair libraries for two *P. rumanzovia* samples: one homozygous simple morph female and one homozygous white morph female. We size-selected DNA using the BluePippin platform (Sage Science) and constructed 3 kb mate-pair libraries using the Nextera Mate Pair Library Prep Kit (Illumina). We assembled the combined dataset of 100 bp paired-end and 3 kb mate-pair libraries for each individual using Platanus^43^. With each assembly, we then used BLAST^44^ and BLAT^45^ to find scaffolds containing *dsx* exons, using the *P. polytes dsx* exons as the queries. Once we had identified the relevant scaffolds we aligned them using MAFFT^46^ and calculated sequence identity between the aligned regions with sliding 100 bp windows in Geneious^47^.

### Genome-wide and *dsx* phylogeny estimation

Approximately 3.4 million SNP calls from genome-wide coding sequence for 50 high quality individuals representing 4 polymorphic species and 16 monomorphic species were aligned in Geneious^47^ and converted to PHYLIP format. We inferred a genome-wide maximum-likelihood tree using the GTRGAMMA model with 100 bootstraps in RAxML^48^. The RAxML output was uploaded to iTOL^49^ to create the tree image.

We phased 6730 SNPs from the *dsx* region for 116 individuals representing 4 polymorphic species and 16 monomorphic species using BEAGLE^50^. We aligned sequences and converted them to PHYLIP format in Geneious^47^ and constructed a maximum-likelihood trees with RAxML^48^ using the GRTGAMMA model and 100 bootstraps. We constructed the tree images by uploading the RAxML outputs to iTOL^49^.

### Gene conversion analysis

We used GENECONV^28^ software to test for gene conversion between *dsx* haplotypes. GENECONV searches for tracts of shared sequence bounded by variable sites between alleles. The software then assesses significance using permutation testing and corrects significance values for multiple comparisons and sequence length. We used a phased full *dsx* region alignment (~100 kb) of all the *dsx* haplotypes from the polymorphic taxa as input and options -Seqtype=SILENT to minimize false positives^51,52^.

### Trans-species SNP analysis

For each species, SNP calls for the *dsx* region were extracted and filtered using VCFTools^34^ option --max-missing 0.75 to include sites were at least 75% of the individuals were genotyped. The filtered SNPs were then manually scored for genotype-wing pattern phenotype associations for each species. Then, R^33^ was used to identify shared SNPs among species.

### Tajima’s D analysis

For each species, we calculated Tajima’s D in 10 kb windows across the genome based on SNP calls using VCFTools^34^ with option --TajimaD 10000. We calculated the distribution of the number of variants per window for each taxon and removed windows in the bottom 10%. Genome-wide Tajima’s D distributions were plotted with the ggplot2 R package^38,40^.

### Reporting summary

Further information on research design is available in the Nature Research Reporting Summary linked to this article.

## Supplementary information

Supplementary Information

Reporting Summary

Description of Additional Supplementary Files

Supplementary Data 1

## Data Availability

Sequence data generated for this study can be found under NCBI BioProject PRJNA589019, (see Supplementary Data 1 for accession numbers). The *Papilio polytes* data used in this study can be found under NCBI BioProject PRJNA234541, (SRR1118152, SRR1118150, SRR1118145, SRR1112619, SRR1112070, SRR1111718). The *Papilio xuthus* reference genome can be found under NCBI BioProject PRJDB2956.

## Code availability

All custom codes are available from the authors upon request.
