# Supplementary material for: A shared genetic basis of mimicry across swallowtail butterflies points to ancestral co-option of doublesex
Source: Nat Commun. 2020 Jan 3;11:6. doi: 10.1038/s41467-019-13859-y (PMC6941989; doi:10.1038/s41467-019-13859-y)
Supplement: Supplementary file 1 — Supplementary Information [file 41467_2019_13859_MOESM1_ESM.pdf]

Supplementary Information

A shared genetic basis of mimicry across swallowtail butterflies points to ancestral co-option of *doublesex*

Palmer & Kronforst

Supplementary Note 1

Microsporidian sequences in the *Papilio xuthus* genome

To assess whether microsporidian sequences in the *P. xuthus* genome assembly impacted our results, we first blasted the 5572 *P. xuthus* scaffolds to the *N. bombycis* genome (GCA_000383075.1). We identified 914 *P. xuthus* scaffolds with blast hits to the *N. bombycis* genome. There were no blast hits in *dsx*, with the nearest surrounding hits upstream or downstream 233kb and 133kb away, respectively. We then analyzed our SNP datasets to assess how many SNPs fell within regions of blast hits between *P. xuthus* and *N. bombycis*. The results are summarized in Supplementary Table 2. Although the *P. rumanzovia*, *P. aegeus*, and 20-species coding sequence datasets showed negligible numbers of potentially microsporidian variants, the *P. memnon* dataset showed more SNPs in regions of potentially microsporidian origin. This could indicate that the *P. memnon* individuals used in this study carried microsporidian parasites that were captured in our sequence data. We generated a new manhattan plot for the *P. memnon* GWAS (Supplementary Figure 3), and verified that variants in putative microsporidian regions did not affect our results.

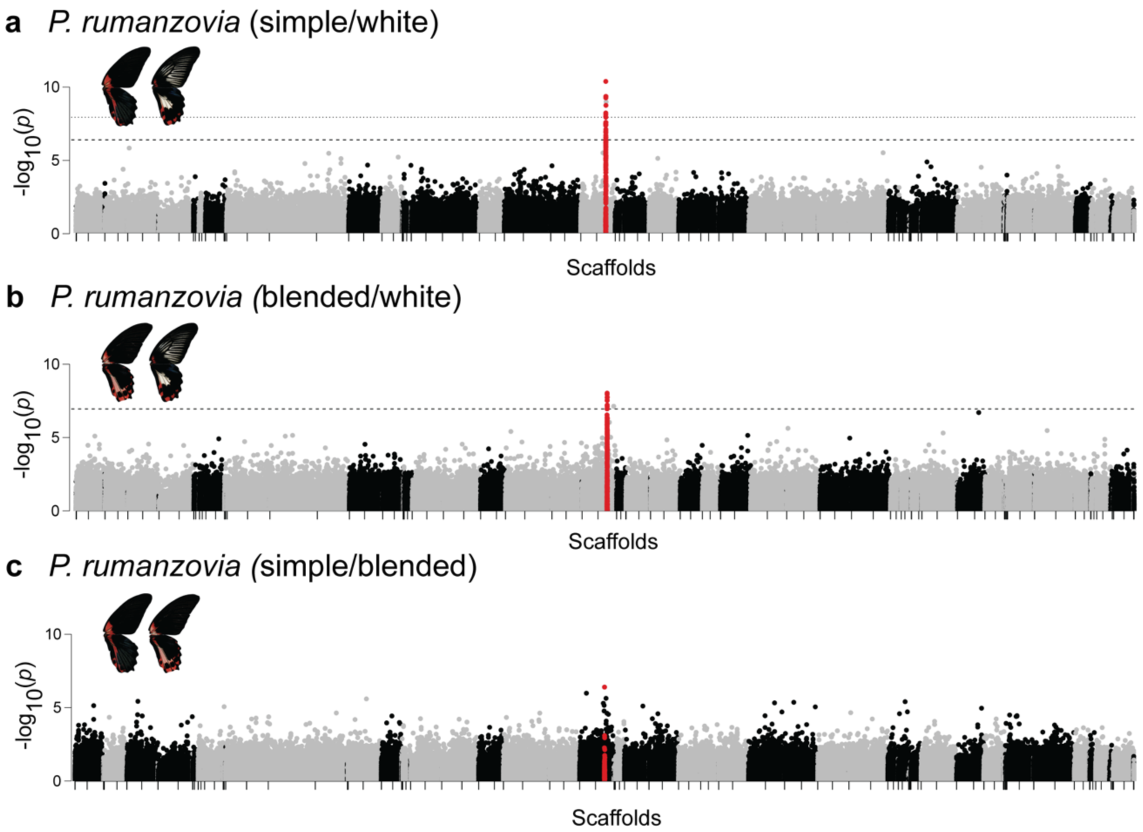

Supplementary Figure 1 Pairwise genome-wide association studies (GWAS) for *P. rumanzovia*. **a** Simple morph vs. white morph GWAS. **b** Blended morph vs. white morph GWAS. **c** Simple morph vs. blended morph GWAS. Red points indicate SNPs in the *dsx* region. Dashed and dotted lines show the false discovery rate (q value) cutoffs of 0.01 and 0.001, respectively. The q value lines could not be drawn for **c** because the lowest q value observed was 0.31.

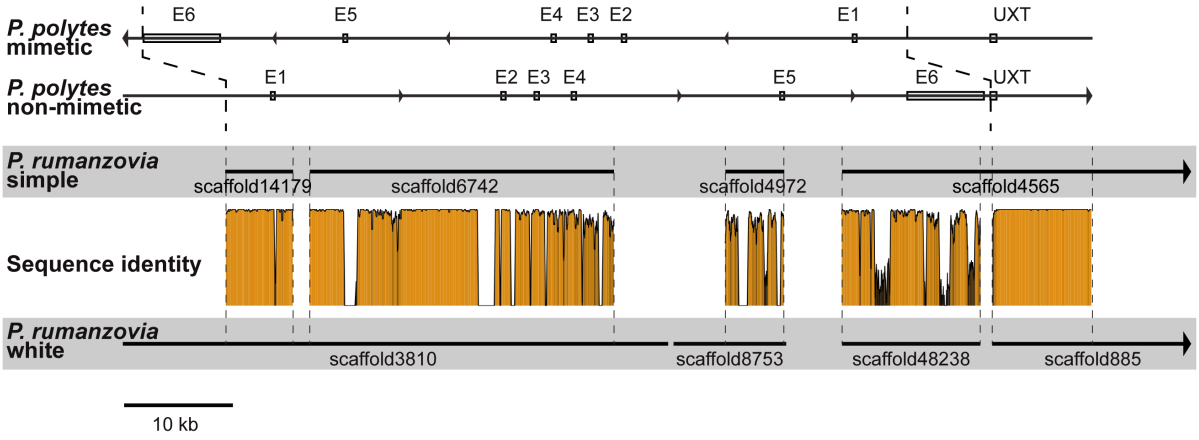

Supplementary Figure 2 Schematic of the *P. polytes dsx* region and scaffolds containing *dsx* exons from *P. rumanzovia* simple and white morph *de novo* assemblies. Sequence identity is shown between homologous *P. rumanzovia* scaffolds based on sliding 100bp windows. E1-6 denote *dsx* exons and UXT denotes the coding sequence of the neighboring gene *ubiquitously expressed transcript*. The physical orientation is shown to match the orientation shown in earlier figures.

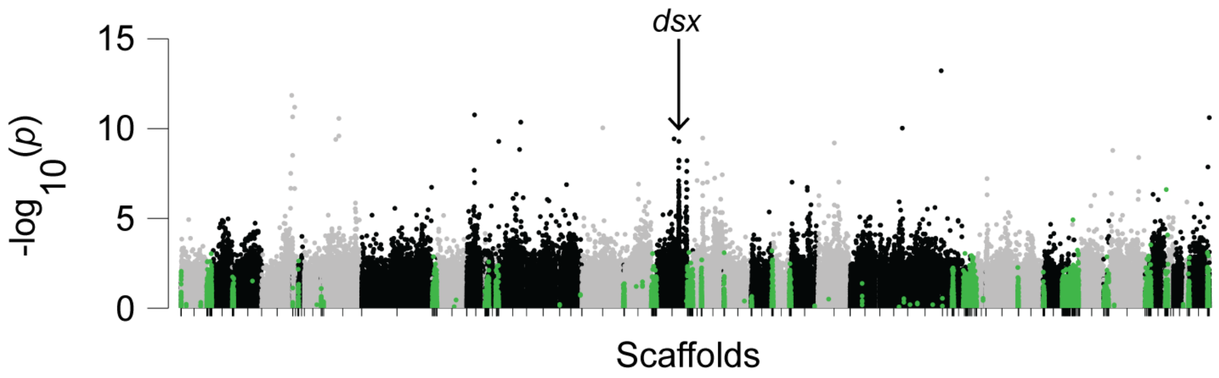

Supplementary Figure 3 *P. memnon* GWAS. SNPs highlighted in green occur in putative microsporidian sequences within the reference genome.

Supplementary Table 1 Putative gene conversion tracts between *dsx* haplotypes.

**Species**	**Morphs**	**Sim P value**[a]	**BC KA P value**[b]	**Start (bp)**	**End (bp)**	**Length**
P. polytes	Mimetic/ non-mimetic	0.0029	0.12122	5148362	5160925	12564
P. rumanzovia	Simple/white	0.0167	0.50317	5090846	5118037	27192

[a] Sim P value based on 10,000 permutations

[b] Bonferroni-corrected Karlin-Altschul P value

Supplementary Table 2 Variants within putative microsporidian sequences within the *P. xuthus* reference genome.

**Dataset analyzed**	**Total number of variants**	**Number of variants in putative microsporidian regions within the *P. xuthus* genome assembly**
P. rumanzovia GWAS	869,047	52
P. memnon GWAS	1,808,282	26,489
P. aegeus PCA	1,605,509	124
20-species genome-wide coding sequence SNPs	3,433,194	5,083
