# Supplementary material for: A shared genetic basis of mimicry across swallowtail butterflies points to ancestral co-option of doublesex
Source: Nat Commun. 2020 Jan 3;11:6. doi: 10.1038/s41467-019-13859-y (PMC6941989; doi:10.1038/s41467-019-13859-y)
Supplement: Supplementary file 2 — Reporting Summary [file 41467_2019_13859_MOESM2_ESM.pdf]

nature research

Corresponding author(s): Daniela H Palmer

Last updated by author(s): Nov 29, 2019

Reporting Summary

Nature Research wishes to improve the reproducibility of the work that we publish. This form provides structure for consistency and transparency in reporting. For further information on Nature Research policies, see Authors & Referees and the Editorial Policy Checklist.

Statistics

For all statistical analyses, confirm that the following items are present in the figure legend, table legend, main text, or Methods section.

n/a	Confirmed	
☐	☒	The exact sample size (*n*) for each experimental group/condition, given as a discrete number and unit of measurement
☒	☐	A statement on whether measurements were taken from distinct samples or whether the same sample was measured repeatedly
☐	☒	The statistical test(s) used AND whether they are one- or two-sided<br>*Only common tests should be described solely by name; describe more complex techniques in the Methods section.*
☒	☐	A description of all covariates tested
☐	☒	A description of any assumptions or corrections, such as tests of normality and adjustment for multiple comparisons
☒	☐	A full description of the statistical parameters including central tendency (e.g. means) or other basic estimates (e.g. regression coefficient) AND variation (e.g. standard deviation) or associated estimates of uncertainty (e.g. confidence intervals)
☒	☐	For null hypothesis testing, the test statistic (e.g. *F*, *t*, *r*) with confidence intervals, effect sizes, degrees of freedom and *P* value noted<br>*Give P values as exact values whenever suitable.*
☒	☐	For Bayesian analysis, information on the choice of priors and Markov chain Monte Carlo settings
☒	☐	For hierarchical and complex designs, identification of the appropriate level for tests and full reporting of outcomes
☒	☐	Estimates of effect sizes (e.g. Cohen's *d*, Pearson's *r*), indicating how they were calculated

Our web collection on statistics for biologists contains articles on many of the points above.

Software and code

Policy information about availability of computer code

Data collection	Illumina software on HiSeq 2500 and HiSeq 4000 sequencing instruments. Built-in software on BluePippin instrument.
Data analysis	Trimmomatic 0.36<br>Bowtie2 2.1.0<br>Picard 1.125<br>GATK 3.8.0<br>Plink 1.90<br>VCFTools 0.1.14<br>GEMMA 0.94<br>R 3.6.1<br>Haploview 4.2<br>Platanus 1.2.1<br>Blastn 2.6.0<br>BLAT 35<br>MAFFT 1.3.6<br>Geneious R9<br>RAxML 8<br>iTOL v3<br>BEAGLE 4.0<br>GENECONV 1.81a

For manuscripts utilizing custom algorithms or software that are central to the research but not yet described in published literature, software must be made available to editors/reviewers. We strongly encourage code deposition in a community repository (e.g. GitHub). See the Nature Research guidelines for submitting code & software for further information.

Data

Policy information about availability of data

All manuscripts must include a data availability statement. This statement should provide the following information, where applicable:

- Accession codes, unique identifiers, or web links for publicly available datasets
- A list of figures that have associated raw data
- A description of any restrictions on data availability

Sequence data generated for this study can be found under BioProject PRJNA589019, [http://www.ncbi.nlm.nih.gov/bioproject/589019] (see Supplementary Data 1 for accession numbers). The Papilio polytes data used in this study can be found under BioProject PRJNA234541, [https://www.ncbi.nlm.nih.gov/bioproject/234541] (SRR1118152 [https://www.ncbi.nlm.nih.gov/sra/?term=SRR1118152], SRR1118150 [https://www.ncbi.nlm.nih.gov/sra/?term=SRR1118150], SRR1118145 [https://www.ncbi.nlm.nih.gov/sra/?term=SRR1118145], SRR1112619 [https://www.ncbi.nlm.nih.gov/sra/?term=SRR1112619], SRR1112070 [https://www.ncbi.nlm.nih.gov/sra/?term=SRR1112070], SRR1111718 [https://www.ncbi.nlm.nih.gov/sra/?term=SRR1111718]). The Papilio xuthus reference genome can be found under BioProject PRJDB2956 [https://www.ncbi.nlm.nih.gov/bioproject/PRJDB2956/].

Field-specific reporting

Please select the one below that is the best fit for your research. If you are not sure, read the appropriate sections before making your selection.

☐ Life sciences ☐ Behavioural & social sciences ☒ Ecological, evolutionary & environmental sciences

For a reference copy of the document with all sections, see nature.com/documents/nr-reporting-summary-flat.pdf

Ecological, evolutionary & environmental sciences study design

All studies must disclose on these points even when the disclosure is negative.

Study description	Butterfly DNA was sequenced and assembled/aligned to a reference genome. Population genetic measures were taken and phylogenetic relationships were inferred.
Research sample	A group of Papilio butterflies were sequenced, and existing data for Papilio polytes was downloaded from SRA (see Data Availability statement). Supplementary Data 1 shows detailed information for each sample in the study. These butterflies were chosen because they show variability in polymorphic wing patterning.
Sampling strategy	Samples were chosen to maximize the wing pattern diversity included in the study.
Data collection	Data collection consisted of whole-genome sequencing, which was conducted by staff at the University of Chicago Functional Genomics Facility.
Timing and spatial scale	n/a
Data exclusions	No data were excluded from analyses.
Reproducibility	We did not attempt to repeat any analyses.
Randomization	No randomization was performed.
Blinding	n/a

Did the study involve field work? ☐ Yes ☒ No

Reporting for specific materials, systems and methods

We require information from authors about some types of materials, experimental systems and methods used in many studies. Here, indicate whether each material, system or method listed is relevant to your study. If you are not sure if a list item applies to your research, read the appropriate section before selecting a response.

Materials & experimental systems

n/a	Involved in the study
☒	☐ Antibodies
☒	☐ Eukaryotic cell lines
☒	☐ Palaeontology
☐	☒ Animals and other organisms
☒	☐ Human research participants
☒	☐ Clinical data

Methods

n/a	Involved in the study
☒	☐ ChIP-seq
☒	☐ Flow cytometry
☒	☐ MRI-based neuroimaging

Animals and other organisms

Policy information about studies involving animals; ARRIVE guidelines recommended for reporting animal research

Laboratory animals	The study did not involve lab animals.
Wild animals	Butterflies were purchased from insect collectors or commercial butterfly farms. Supplementary Data 1 shows all information on species, sex, and location of origin for each sample.
Field-collected samples	See above.
Ethics oversight	No ethical approval was necessary as all samples were invertebrates.

Note that full information on the approval of the study protocol must also be provided in the manuscript.
