# Supplementary material for: A shared genetic basis of mimicry across swallowtail butterflies points to ancestral co-option of doublesex
Source: Nat Commun. 2020 Jan 3;11:6. doi: 10.1038/s41467-019-13859-y (PMC6941989; doi:10.1038/s41467-019-13859-y)
Supplement: Supplementary file 3 — Description of Additional Supplementary Files [file 41467_2019_13859_MOESM3_ESM.pdf]

Description of Additional Supplementary Files

File Name: Supplementary Data 1
Description: Sample information and sequencing statistics.
